# Construction and characterization of a *Saccharomyces cerevisiae* strain able to grow on glucosamine as sole carbon and nitrogen source

**DOI:** 10.1038/s41598-018-35045-8

**Published:** 2018-11-16

**Authors:** Carmen-Lisset Flores, Carlos Gancedo

**Affiliations:** Instituto de Investigaciones Biomédicas “Alberto Sols” CSIC-UAM. Calle Arturo Duperier 4, 28029 Madrid, Spain

## Abstract

*Saccharomyces cerevisiae* can transport and phosphorylate glucosamine, but cannot grow on this amino sugar. While an enzyme catalyzing the reaction from glucosamine-6-phosphate to fructose-6-phosphate, necessary for glucosamine catabolism, is present in yeasts using N-acetylglucosamine as carbon source, a sequence homology search suggested that such an enzyme is absent from *Saccharomyces cerevisiae*. The gene *YlNAG1* encoding glucosamine-6-phosphate deaminase from *Yarrowia lipolytica* was introduced into *S. cerevisiae* and growth in glucosamine tested. The constructed strain grew in glucosamine as only carbon and nitrogen source. Growth on the amino sugar required respiration and caused an important ammonium excretion. Strains overexpressing *YlNAG1* and one of the *S. cerevisiae* glucose transporters *HXT1, 2*, 3, *4, 6* or 7 grew in glucosamine. The amino sugar caused catabolite repression of different enzymes to a lower extent than that produced by glucose. The availability of a strain of *S. cerevisiae* able to grow on glucosamine opens new possibilities to investigate or manipulate pathways related with glucosamine metabolism in a well-studied organism.

## Introduction

Glucosamine (2-amino-2-deoxy-glucose) is found in nature as a component of polysaccharides and in glycosylated proteins or lipids. It is abundant in soils as a product of degradation of the chitin present in fungal cell walls^1^. Glucosamine has attracted much attention due to its multiple effects on mammals, in which it can lead to insulin resistance in adipocytes or skeletal muscle, both in cultured cells or *in vivo*^2,3^. In addition, it is being used for the treatment of symptomatic joint pain in humans^4,5^ although there are conflicting reports on its efficacy^6^. Glucosamine has also been reported to increase the life span of *Caenorhabditis elegans* and ageing mice through glycolysis impairment^7^. A participation of the amino sugar in the recovery from desiccation of *Chironomus ramosus* larvae has been recently discovered^8^.

Some microorganisms can use glucosamine as carbon source^9,10^ and its metabolism has been studied in detail in *Escherichia coli*^11,12^. Among yeasts, the use of glucosamine as carbon source is not widespread and has been described only for a few species^13^. In the model yeast *Saccharomyces cerevisiae* glucosamine does not support growth as carbon source, although it is transported into the cell and phosphorylated by hexokinase^14–16^. In this yeast glucosamine acts as a non-metabolizable glucose analogue and, as such, it has been used to vary the steady-state growth rate of populations of *S. cerevisiae*^17^ and to obtain mutants insensitive to catabolite repression by selecting for growth on carbon sources other than glucose in the presence of glucosamine^18–22^. Most of those mutants presented Mendelian monogenic inheritance but some of them showed a non-Mendelian, cytoplasmic inheritance^18^. More recently, Brown and Lindquist^23^ isolated *S. cerevisiae* mutants able to grow on glycerol in the presence of 0.05% glucosamine and determined that the resistance to glucosamine implicated the appearance of a prion that they named [*GAR*^+^].

It appeared of interest to determine why *S. cerevisiae* is unable to use glucosamine as carbon source and to try to generate strains able to use it, in order to study their behaviour and also in view of a possible industrial utilization. We have addressed this question and we describe in this article the construction of a *S. cerevisiae* strain able to use glucosamine for growth as sole carbon and nitrogen source, as well as some physiological properties of this strain.

## Results

### *S. cerevisiae* lacks glucosamine-6-P deaminase

**Figure 1:**
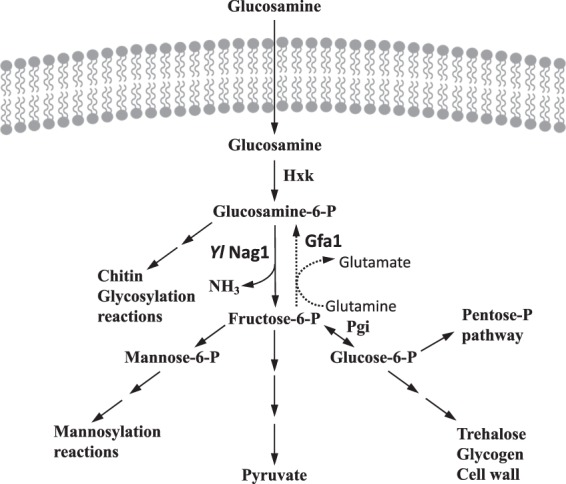
Pathway of glucosamine utilization. Glucosamine is phosphorylated by hexokinase, Hxk, deaminated and isomerized by glucosamine-6-P deaminase, Nag1, and enters the glycolytic pathway. (*Yl* indicates *Y. lipolytica*, the source of the enzyme used in this work). The importance of different hexose-monophosphates as starting compounds for different reactions is indicated. Gfa1 indicates glutamine-fructose-6-phosphate amidotransferase that catalyzes the reaction opposite to the one catalyzed by Nag1 (dotted line).

Metabolism of glucosamine occurs in most organisms through the pathway depicted in Fig. 1. The sugar is transported inside the cell, and phosphorylated to glucosamine-6-phosphate. Entrance into the glycolytic pathway occurs after its deamination-isomerization to fructose-6-phosphate by glucosamine-6-P deaminase (EC 3.5.99.6). Since *S. cerevisiae* transports glucosamine^15^ and phosphorylates it to glucosamine-6-phosphate^14,16^ we asked why this yeast was unable to use glucosamine as carbon source. An *in silico* search in the *S. cerevisiae* genome for genes encoding enzymes that participate in glucosamine metabolism showed that no gene encoding a protein with sequence similarity to that of glucosamine-6-P deaminase had been reported in this yeast.

### Expression of *YlNAG1* in *S. cerevisiae* allows growth in glucosamine

Glucosamine-6-P deaminase is present in yeasts that use N-acetylglucosamine such as *Candida albicans*^24^. In *Yarrowia lipolytica* a gene, *YALI0C01419g*, putatively encoding such a protein has also been identified and named *YlNAG1*^25^. The putative protein encoded by this gene has a sequence of 273 amino acids with up to 60% identity with proteins annotated as glucosamine-6-P deaminase from other yeasts and fungi. We assayed the activity of glucosamine-6-P deaminase in *Y. lipolytica* grown in N-acetylglucosamine and found a specific activity of 270 mU/mg protein, while it was barely detectable during growth in glucose. The low enzymatic activity in glucose is in agreement with the low amounts of the corresponding mRNA in this condition^25^. We determined the Km value of the enzyme for glucosamine-6-P in dialyzed extracts from *Y. lipolytica* to be 0.4 mM.

**Figure 2:**
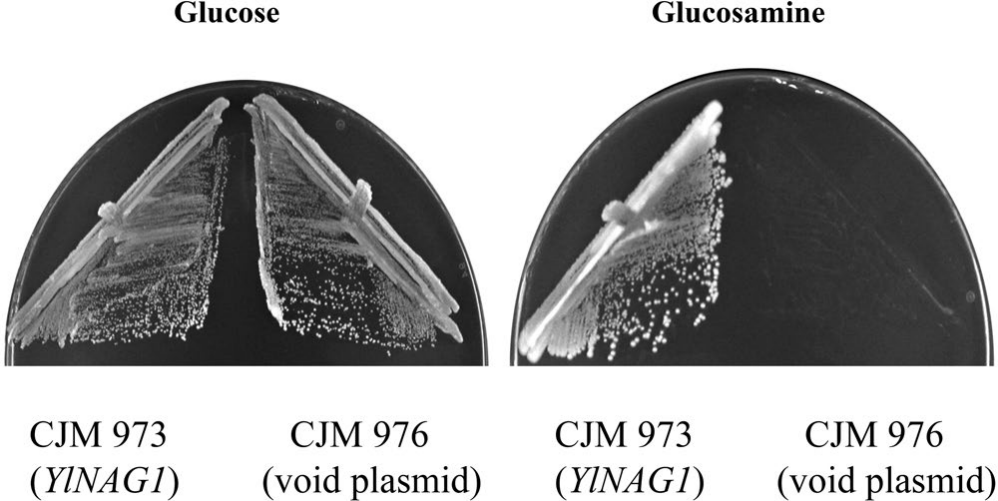
Growth on glucosamine depends on the presence of *YlNAG1*. Strains CJM973 with plasmid pCL257 with the gene *YlNAG1* encoding glucosamine-6-P deaminase, and CJM976, with a similar plasmid without *YlNAG1* were grown with the indicated carbon sources as described in Methods. The plates were grown for 3 days at 30 °C.

We cloned the gene *YlNAG1* in a *S.cerevisiae* multicopy plasmid as described in Methods and transformed a strain of *S. cerevisiae* with it. Transformants were selected in glucose by uracil prototrophy and screened for growth on plates with glucosamine as carbon source. We observed that the transformants could use glucosamine both as carbon and nitrogen source and all subsequent experiments were done in these conditions. Figure 2 shows the behaviour of one of such transformants (CJM973, Table 1). As can be seen the transformed strain bearing the *YlNAG1* gene grew on glucosamine while a control with a void plasmid did not. Loss of the plasmid also abolished growth on glucosamine indicating that expression of *YlNAG1* conferred the capacity to grow in glucosamine. Expression of *YlNAG1* was the only requirement to grow on glucosamine as demonstrated by the following experiment. Strain CLF312 (Table 1) was transformed with plasmid pCL313 (see Methods) to integrate the *YlNAG1* gene in the *S. cerevisiae* genome at the *LEU2* locus. The integration at the correct site regenerated a functional *LEU2* gene (strain CJM3083). Two positive independent transformants growing in glucosamine and being leucine prototrophs were crossed with strain CLF307 (Table 1) and tetrads dissected. In 8 complete tetrads derived from each cross, growth in glucosamine and leucine prototrophy cosegregated in a 2+: 2− fashion indicating that growth in this sugar was due to the presence of the *YlNAG1* gene at the *LEU2* locus.

**Table 1:** *S. cerevisiae* strains used in this work. Plasmids pCL257, pCL258 and p426 are described in detail in the Methods section.

Strain	Relevant characteristics	Origin/Reference
CJM 534	W303 background *ade2 his3 trp1 ura3*	J. M. Gancedo^72^
CJM 973	CJM534/pCL257 (*YlNAG1*)	This work
CLF312	W303 background *leu2 ura3*	This work
CJM3083	*YlNAG1* integrated into *LEU2* locus of CLF312	This work
CLF307	W303 background *leu2 trp1*	This work
CJM3095	Respiratory deficient (*petite*) strain derived from CJM973	This work
CJM 278 (originally RE700A)	*hxt1∆::HIS3::∆hxt4 hxt5::LEU2 hxt2∆::HIS3 hxt3∆::LEU2::∆hxt6 hxt7::HIS3 ura3*	E. Boles^31^
CJM 3070 (Originally EBY.VW4000)	Δ *hxt1-17* Δ *gal2* Δ*snf3* Δ *stl1* Δ *agt1* Δ *mph2* Δ *mph3 his3 leu2 trp1 ura3*	E. Boles^33^
CJM 3071	CJM3070/pCL258 (*YlNAG1*)	This work
CJM 3132	CJM3071/p426 *HXT1*	This work
CJM 3131	CJM3071/p426 *HXT2*	This work
CJM 3111	CJM3071/p426 *HXT3*	This work
CJM 3080	CJM3071/p426 *HXT4*	This work
CJM 3100	CJM3071/p426 *HXT5*	This work
CJM 3112	CJM3071/p426 *HXT6*	This work
CJM 3136	CJM3071/p426 *HXT7*	This work
CJM 3113	CJM 3071/p426	This work

The doubling time in glucosamine of the strains carrying the gene *YlNAG1* either in a multicopy plasmid or integrated in the genome was greater than that in glucose. In glucose-ammonium medium the doubling time was 152 ± 4 minutes (mean ± standard deviation of four independent cultures) while in glucosamine the doubling times were 238 ± 9 minutes for the strain with the multicopy plasmid (mean ± standard deviation of four independent cultures) and 380 ± 10 minutes for the strain with the integrated gene (mean ± standard deviation of three independent cultures). The assayed glucosamine-6-P deaminase activity was 98 ± 9 mU/mg protein in the strain with the multicopy plasmid and 28 ± 4 mU/mg protein in the strain carrying the gene integrated in the genome (means ± standard deviation of three independent biological samples). The Km of glucosamine-6-P deaminase for glucosamine-6-P assayed in dialyzed extracts from *S. cerevisiae* was similar to that found when the enzyme was assayed in extracts from *Y. lipolytica*. The enzyme was not activated by N-acetylgucosamine-6-P in contrast with the enzyme from *E. coli*^26^.

### Glucosamine metabolism requires respiration

**Figure 3:**
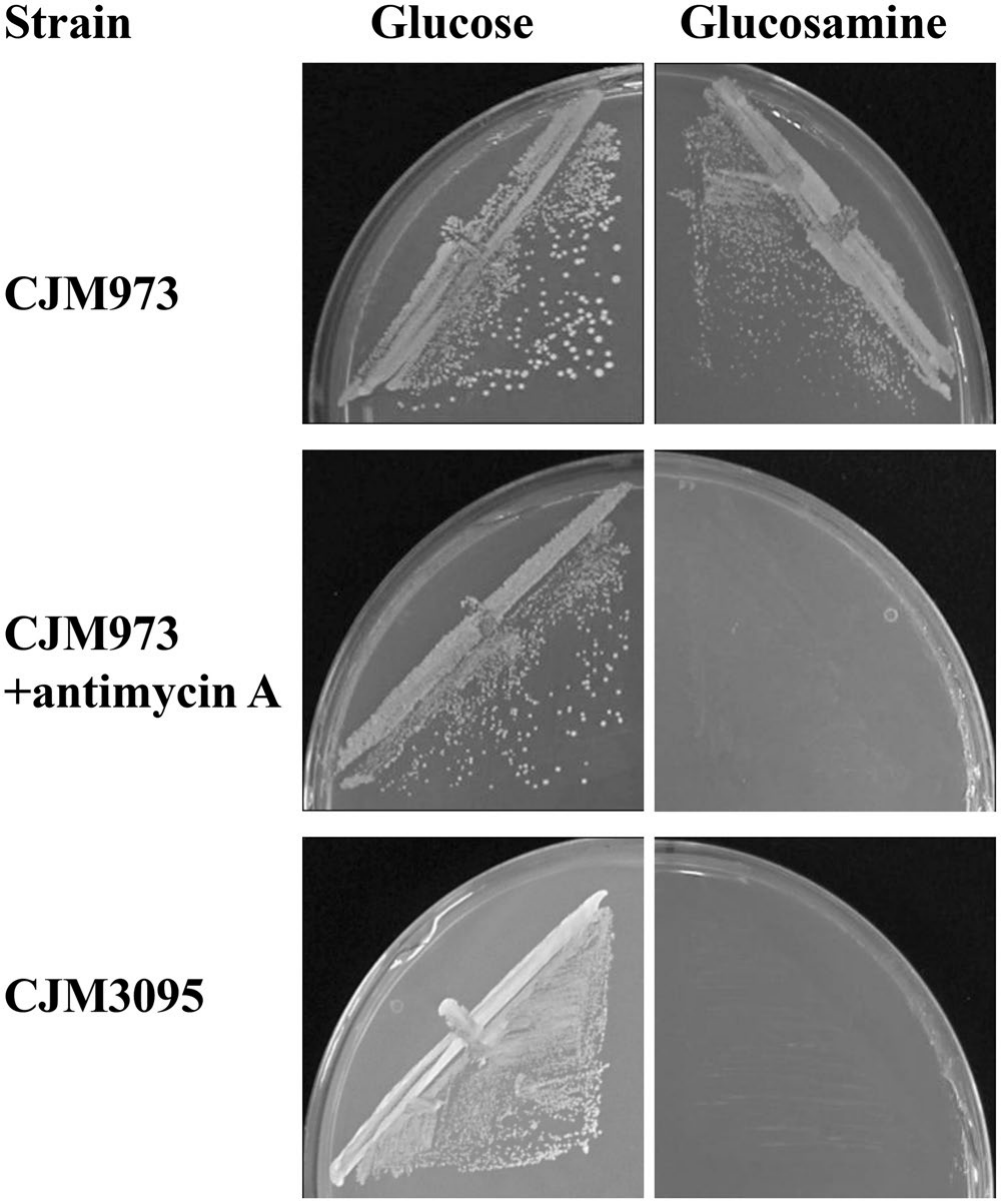
Growth on glucosamine depends on respiration. Strain CJM973 carries plasmid pCL257 with the gene *YlNAG1* encoding glucosamine-6-P deaminase. CJM 3095 is a respiratory deficient strain derived from CJM 973 obtained as described in Methods. Plates with different carbon sources and the indicated addition were streaked and incubated for 3 days at 30 °C except for CJM3095 that was incubated for 5 days.

*S. cerevisiae* can grow in certain sugars either by fermenting them to ethanol or by oxidizing them through the citric acid cycle to CO_2_. Glucosamine enters the glycolytic pathway at the level of fructose-6-phosphate, which can follow the fermentative or the oxidative pathway. The inhibition of the oxidative pathway with antimycin A that blocks the transfer of electrons between cytochrome b and cytochrome c precluded growth on glucosamine but not on glucose (Fig. 3). To discard a possible effect of the drug unrelated with respiration, a respiratory deficient strain (CJM 3095) was derived from the CJM973 strain and its growth tested on glucosamine. CJM3095 did not grow on glucosamine (Fig. 3) indicating that the inhibition of growth by antimycin A is not an indirect effect of the drug, but a consequence of the block of respiration. Both results are consistent with the requirement of a functional respiratory pathway for growth in the amino sugar. It should be noted, however, that a significant proportion of the glucosamine consumed during growth (*ca*. 30%) was transformed in ethanol.

### Growth in glucosamine causes excretion of ammonium

**Figure 4:**
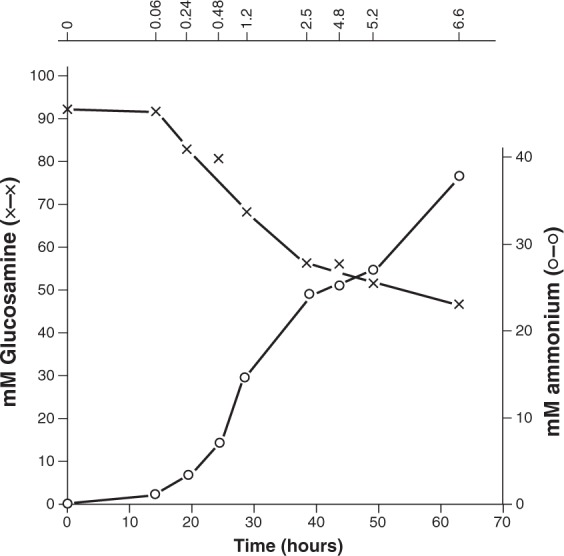
Production of ammonium during growth in glucosamine. Strain CJM973 was grown at 30 °C in a minimal medium with glucosamine as carbon and nitrogen source as described in Methods. Samples were taken along time, centrifuged and glucosamine and ammonium were determined in the supernatant as described in Methods. The upper horizontal bar indicates the OD of the culture when samples were taken.

We assayed glucosamine consumption and biomass production in cultures of the constructed strain growing in this sugar and obtained a biomass yield of 190 mg dw yeast/g glucosamine consumed. 1 g glucosamine contains 78 mg nitrogen. Based on the biomass composition of yeast^27,28^ a yeast molar biomass formula can be written as C_3.8_ N_0.61_ O_2.1_ H_7_^29^, therefore 190 mg dry yeast contain only 17 mg of nitrogen. This shows that the yeast growing in glucosamine has taken in an excess of nitrogen that needs to be discarded. This excess will be initially in the form of ammonium produced by deamination of glucosamine-6-P but since excess ammonium is toxic, the yeast needs to get rid of it. We determined the ammonium concentration in a culture medium of the yeast growing in glucosamine along time (Fig. 4). As it can be seen glucosamine disappearance and ammonium appearance follow parallel inverted kinetics, showing that the yeast excretes ammonium to get rid of an excess of this compound. A control culture without yeast showed that glucosamine was stable along the time assayed and therefore did not contribute to the detected ammonium. We have found that *Pichia cactophila*, a yeast naturally able to grow in glucosamine, also excreted ammonium when growing in this sugar; ca. 16 mM at an OD of 4.5 (three independent cultures), a value similar to that measured in *S. cerevisiae*.

Hess *et al*.^30^ found that under potassium limitation ammonium became toxic for *S. cerevisiae*. Under this condition detoxification of ammonium excess was achieved by excretion of amino acids to the medium. We therefore analysed if the constructed yeast growing in glucosamine also excreted amino acids to the medium. The total concentration detected by LC/MS was less than 0.5 mM.

### Identification of glucosamine transporters

*S. cerevisiae* possesses an array of about 20 hexose transporters, seven of which are metabolically important for glucose transport^31,32^. To determine which of the hexose transporters were important for glucosamine transport we transformed a yeast strain devoid of transporters Hxt1 through Hxt7 (CJM 278, Table 1) with plasmid pCL257 carrying *YlNAG1* and tested the growth in glucosamine. The absence of growth indicated that glucosamine was taken up by a transporter of this group. Therefore we constructed yeast strains that overexpressed individually transporters Hxt1, 2, 3, 4, 5, 6 and 7 in strain CJM 3070 devoid of the 20 transporters implicated in hexose transport^33^. Several transporters supported growth in glucosamine but Hxt7, 4 and 2 were more effective (Table 2). We also found that overexpression of *HXT5* did not support growth on this amino sugar.

**Table 2:** Growth in glucosamine of strains overexpressing single glucose transporters.

Strain/Transporter	Dry weight (mg/ml culture)
CJM 973/Wild type	1.8 ± 0.3
CJM 3132/*HXT1*	0.4 ± 0.1
CJM 3131/*HXT2*	0.9 ± 0.2
CJM 3111/*HXT3*	0.7 ± 0.2
CJM 3080/*HXT4*	0.9 ± 0.1
CJM 3100/*HXT5*	≤0.1
CJM 3112/*HXT6*	0.6 ± 0.2
CJM 3136/*HXT7*	1.1 ± 0.3

The indicated yeast strains were grown in glucose, washed with water and inoculated in media with glucosamine as carbon and nitrogen source (see Methods) to an initial OD of 0.05. After growing for 72 h the cultures were filtered and dry weight determined as indicated in Methods. A culture with strain CJM 3113 carrying an empty plasmid was run in parallel and the value of its dry weight was subtracted from that of the other cultures. Figures are mean values followed by their standard deviation from at least three independent cultures.

While strains overexpressing genes *HXT1, 2* or 3 grew in glucosamine, strains expressing individually these genes from their chromosomal copy did not.

### Effect of glucosamine on catabolite repression

**Figure 5:**
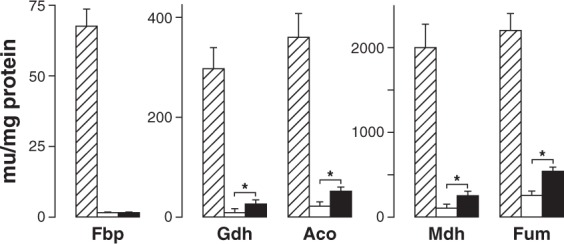
Effect of glucosamine on catabolite repression. Strain CJM973 was grown in media with ethanol as a control of derepression (dashed columns), glucose (white columns) or glucosamine (black columns). Activities of the indicated enzymes were measured in extracts from exponentially growing cultures as described in Methods. Fbp, fructose-1,6-biphosphatase; Gdh, glutamate dehydrogenase (NAD); Aco, aconitase; Mdh, malate dehydrogenase; Fum, fumarase. The values of activity are the mean of five independent experiments with the error bars indicating the standard deviation. Statistical significance was determined using the t-test (*p < 0.05).

In a wild-type *S. cerevisiae* strain glucosamine impairs growth in different carbon sources and this has been ascribed to a capacity of glucosamine to cause catabolite repression^19,20^. To compare the effects of glucosamine and glucose on catabolite repression we assayed the activities of several enzymes subject to this regulatory mechanism in the strain carrying *YlNAG1* grown in these sugars and compared them with those found in ethanol, a carbon source in which those enzymes are derepressed. The results are shown in Fig. 5. Glucosamine repressed all the enzymes tested but except for fructose-1,6-bisphosphatase the activities of the enzymes were significantly higher in glucosamine than in glucose cultures (one-tailed, unpaired t-test, n = 5, p < 0.05).

## Discussion

The results presented show that the absence of growth of *S. cerevisiae* in glucosamine is due to the lack of glucosamine-6-P deaminase and that expression of the gene *YlNAG1* from *Y. lipolytica*, which encodes that enzyme, allows growth of *S. cerevisiae* in glucosamine as sole carbon and nitrogen source. Although there is a gene named *NAG1* in *S. cerevisiae*, this designation corresponds to the **N**ested **A**ntisense **G**ene, *YGR031C-A*, which encodes a protein implicated in cell wall function^34^ and not to a gene involved in glucosamine metabolism. The glucosamine-6-P deaminase from *Y. lipolytica* presents an apparent Km towards glucosamine-6-P in the same range as that of homologous enzymes and is not activated by N-acetylglucosamine-6P. A sequence analysis shows that it lacks the reported signature for binding the allosteric effector^35^ as it is also the case for the protein from *C. albicans* that is not activated by N-acetylglucosamine-6P^24^.

Glucosamine-6-P deaminase is present in many different organisms but is absent from plants^36^. Its absence in *S. cerevisiae* is the cause of the toxicity of glucosamine for this yeast, since its transport^15^ and posterior phosphorylation by hexokinase^14,16^ causes an ATP sink and produces accumulation of amino sugar phosphates that might be toxic. Although some bacteria can use glucosamine through an Entner-Doudoroff pathway via glucosaminate to 2-keto-3-deoxy-D-gluconate^9^ for most organisms using glucosamine, glucosamine-6-P deaminase is required. In fact, the activity of this enzyme may limit the flux through the pathway as shown in *E. coli*, where an increase in the amount of the deaminase causes an increase in the growth rate on glucosamine^11^. This is also the case of the *S. cerevisiae* strains constructed in this work. The strain with the multicopy plasmid carrying the *YlNAG1* gene presents a higher deaminase activity and grows faster in glucosamine than the strain with this gene integrated in the genome. Similarly in *Corynebacterium glutamicum* growth on glucosamine occurred only when a mutation in the promoter region of the *nagAB-scrB* operon increased the expression of the gene encoding glucosamine-6-P deaminase^10^.

In yeasts which use N-acetylglucosamine, glucosamine-6-P deaminase is involved in the utilization pathway of this sugar. However, not all the yeasts which use N-acetylglucosamine can use glucosamine^37^ (our unpublished results). This could be related, among other causes, to a low capacity for glucosamine transport and/or phosphorylation or to the inability of glucosamine to induce efficiently the degradation pathway as it happens in *E. coli*^11^. Therefore the presence of glucosamine-6-phosphate deaminase is not sufficient to predict growth of a yeast in glucosamine. Wendland *et al*.^38^ constructed a strain of *S. cerevisiae* expressing the genes of the N- acetylglucosamine degradation pathway from *C. albicans*. Since we have found that *S. cerevisiae* expressing glucosamine-6-phosphate-deaminase grows in glucosamine, it could be expected that the strain constructed by Wendland *et al*.^38^ would be able to grow in this carbon source.

It is noteworthy that growth on glucosamine of the strain generated in this work requires respiration. This respiratory dependence may be related to the slow rate of growth on glucosamine of this yeast strain. In fact, there are many reports showing that a low transport and/or metabolic capacity results in the obligate respiratory growth of different yeasts. *Kluyveromyces lactis* grows on galactose or raffinose only under respiratory conditions but introduction of additional genes encoding the relevant transporters generates strains able to ferment those sugars^39^. Likewise, fermentative growth of *K. lactis* on maltose is also possible if supernumerary maltose transporters and the maltase encoding gene are overexpressed^39^. It has also been shown that *K. lactis* strains defective in the *RAG1* gene, encoding a glucose transporter, are unable to grow in glucose when respiration is inhibited^40^. In *S. cerevisiae* itself, a low trehalose influx has been suggested as a cause for the strictly respiratory growth of the yeast in this disaccharide^41^. Growth in glucose of a *S. cerevisiae hxk1 hxk2 glk1* mutant expressing a N-acetyl glucosamine kinase, an enzyme with a marginal glucokinase activity, is also dependent on respiration^25^. Even glucose may be completely respired in the strong fermentative yeast *S. cerevisiae* if cultured in a chemostat with limiting glucose in the feed^42^.

In spite of the respiration requirement for growth in glucosamine of the constructed strain a certain proportion of the glucosamine consumed was diverted to ethanol production. This behavior is likely due to the requirement of cytosolic acetylCoA for some biosynthetic pathways that has been demonstrated in *S. cerevisiae* using pyruvate decarboxylase mutants^43^.

The biomass composition of yeast^27,28^ and the measured biomass yield of the strain using glucosamine as the sole carbon and nitrogen source indicated that in this condition the organism has incorporated an excess of nitrogen over that required for biosynthesis. The deaminase produces ammonium, a part of which will be used to generate glutamate or glutamine, the usual fate of ammonium^44,45^. However, these sinks are unlikely to cope with the excess of ammonium that should be disposed of. Ammonium is toxic for animal cells and also for plants that have developed mechanisms to eliminate this compound^46,47^. Also Hess *et al*.^30^ showed that ammonium is toxic for *S. cerevisiae* when cultured in glucose at low potassium concentration and that the yeast detoxified it excreting amino acids. We found that during growth in glucosamine *S. cerevisiae* detoxifies ammonium in a different way, namely by excreting it to the medium. This form of detoxification during growth in glucosamine might be general as shown by the same behaviour of *P. cactophila*. Ammonium excretion could take place through the Ato family of ammonium exporters whose function during the growth of yeast on solid media has been studied^48^.

We have determined that from the different glucose transporters present in *S. cerevisiae*^32^ Hxt1/2/3/4/6/7 supported growth in glucosamine to a different extent while Hxt5 did not. Due to the different kinetic properties of these transporters their different effectiveness with glucose analogues is not surprising. It has been found that growth on xylose of a recombinant *S. cerevisiae* strain is only supported by Hxt4, Hxt5, Hxt7 and Gal2^49^. Similarly, in mammals the glucose transporters GLUT1, 2, and 4 can transport glucosamine, but GLUT2 presents a 20-fold higher affinity for glucosamine than for glucose^50^.

An important consideration is the possibility of the existence of a futile cycle in the constructed strain due to the simultaneous activity of the heterologous glucosamine-6-P deaminase and the endogenous glutamine fructose-6-phosphate aminotransferase (Gfa1) which catalyzes the synthesis of glucosamine-6-P from fructose-6-P and glutamine and starts the biosynthetic hexosamine pathway (Fig. 1). Regulation of Gfa1 is not completely understood in yeast although several potential regulatory mechanisms have been described. The phosphatase Glc7 reduces transcription of the gene *GFA1*^51^ and UDP-N-acetylglucosamine inhibits the activity of the enzyme^52–54^. However, the physiological significance of the inhibition appears unlikely since the concentrations of the inhibitor required to cause 50% inhibition of the *S. cerevisiae* enzyme, between 0.6 and 2.5 mM^52,54^, are much higher than the 34 µM intracellular concentration reported for this yeast^55^. Therefore the existence of a futile cycle in the constructed strain remains an open question.

Glucosamine has been used as a non-metabolizable glucose analogue to obtain mutants affected in catabolite repression. However, activities of enzymes repressed by glucose have not been measured systematically in cells grown in a medium containing glucosamine^18–20^. We have shown that when the strain we constructed was grown in this sugar different enzymes were repressed although to a different extent. Except for fructose-1,6-bisphosphatase, the magnitude of the repression of the enzymes studied was lower than that produced by glucose. Catabolite repression in yeasts is a complex phenomenon^56–58^ since not all the genes whose transcription is repressed by glucose respond to the same regulatory circuits^59^. Deletion of an important regulatory gene of catabolite repression such as *HXK2* affects differently the derepression of fructose-1-6-bisphosphatase or glutamate dehydrogenase^60^. Also derepression of different enzymes is unequally affected by the same glucose analogue as shown in the case of 3-methyl glucose that decreased derepression of fructose-1-6-bisphosphatase and malate dehydrogenase by 50% while it did not affect that of glutamate dehydrogenase or cytochrome oxidase^61^. The difference in the magnitude of repression found in glucosamine grown cultures is consistent with the multiplicity of signalling circuits in catabolite repression. Glucosamine could mimic glucose effectively for a given signalling pathway but produce a weaker signal than glucose in another one.

Part of the glucosamine consumed by cells is incorporated into the hexosamine biosynthetic pathway. The implication of this pathway in the glycosylation of proteins connects the amino sugar in higher organisms to different pathologies ranging from limb and girdle disease^62^ to cancer^63^ or diabetes^64^. The constructed strain represents a new useful tool to investigate or manipulate the hexosamine biosynthetic pathway in a well characterized model organism.

## Methods

### Strains and culture conditions

*S. cerevisiae* strains used in this work are shown in Table 1. The respiratory deficient strain CJM 3095 was obtained from strain CJM 973 with ethidium bromide as described by Kontoyiannis^65^. Growth media contained 0.17% Difco yeast nitrogen base with the addition of different carbon sources (final concentrations: 93 mM glucosamine, 110 mM glucose, 434 mM ethanol). Cultures with glucose and ethanol had 40 mM ammonium sulphate as nitrogen source. The media were buffered with 75 mM MES, 5mMTris and had an initial pH of 6.25. Cultures with ethanol were supplemented with 0.2% yeast extract. Glucosamine hydrochloride was from Carbosynth (Compton-Berkshire,UK). Its contamination by glucose was determined to be ≤0.015%. Auxotrophic requirements were added at a final concentration of 20 μg/ml. Antimycin A was used at 2 µg/ml.

Loss of plasmid(s) from yeast was achieved by growing the corresponding strains in rich medium (1% yeast extract, 2% peptone, 110 mM glucose) for multiple generations and identifying colonies from the population after plating in minimal media with the adequate supplements.

### Nucleic acid manipulations, plasmid constructions

Recombinant DNA manipulations were done by standard techniques. DNA corresponding to the gene *YlNAG1* (*YALI0C01419g* in Genolevures, http:gryc.inra.fr) was obtained by PCR using the oligonucleotides YlNAG1-F and YlNAG1-R (Table S1, Supplementary Material) and genomic DNA from *Yarrowia lipolytica* strain PO1a (originally provided by C. Gaillardin, Grignon, France) as template. The resulting DNA was ligated into plasmid pCRBlunt (ThermoFisher Scientific) and excised with *Eag*I. The 0.9 kb fragment was ligated into pDB20^66^ digested with *Not*I to produce plasmid pCL257. A similar plasmid, pCL258, with *LEU2* as marker was obtained by digestion of pCL257 with *Sal*I and *Hpa*I and replacing the *URA3* marker with the *Sma*I-*Sal*I fragment, containing the *LEU2* marker, from plasmid YDp-L^67^. The integrative plasmid pCL313 was constructed as follows: plasmid YIp351^68^ was digested with *Sma*I-*Xba*I and ligated to the 2.75 kb fragment of pCL257 digested with the same enzymes, carrying the expression cassette for *YlNAG1*.

Plasmids carrying the *S. cerevisiae* genes *HXT1*, *HXT2*, *HXT4* and *HXT5* which encode glucose transporters^32^ were obtained as follows. The corresponding DNAs were amplified by PCR from genomic DNA of the strain W303 using the oligonucleotides indicated in Table S1 (Supplementary Material), cloned into plasmid pGEMTeasy (Promega) and sequenced. After excising them with adequate enzymes they were ligated into the yeast expression plasmid p426 kindly donated by E. Boles (Frankfurt, Germany). This plasmid expresses constitutively the genes under the control of a truncated *HXT7* promoter^49^. Several attempts to clone the genes *HXT3* and *HXT6/7* as described above failed due to extensive plasmid sequence rearrangement during amplification in *E. coli*. Therefore cloning of *HXT3* and *HXT6/7* was done by transforming *S. cerevisiae* with a mixture of plasmid p426 digested with *Sma*I-*Sa*lI and the PCR product containing the ORF corresponding to each glucose transporter elongated with a sequence homologous to the plasmid ends. To elongate the ends of the PCR products containing the ORFs of genes *HXT3* and *HXT6/7*, obtained using primers HXT3-F, HXT3-R and HXT6/7-F, HXT6/7-R, these fragments were used in a second round of PCR with primers HXT3-Fa, HXT3-Ra, HXT6/7-Fb and HXT6/7-Rb (Table S1). Constructions were checked by PCR followed by sequencing.

Yeast transformation was done using the lithium acetate method^69^.

### Extracts and assay of enzyme activities

Yeast cell free extracts for determination of glucosamine-6-P deaminase were prepared by breaking the yeast with glass beads in 50 mM potassium phosphate, 1 mM EDTA, 1 mM DTT pH7.6 in five cycles of 1 min of vortexing and 1 min in ice. The extract was centrifuged at 4 °C for 15 min at 20000 × g and the supernatant was dialyzed for two periods of 50 minutes against a 50-fold volume of extraction buffer. Extracts for determination of other activities were done similarly in 20 mM imidazol/HCl buffer pH7 and used without dialysis.

Glucosamine-6-P deaminase was assayed spectrophotometrically at 340 nm coupling the production of fructose-6-P from glucosamine-6-phosphate to an auxiliary system generating NADPH essentially as in White and Pasternak^70^. The assay mixture contained 0.1 M TRIS/HCl pH7.6, 0.2 mM NADP, 2 units/ml each of phosphoglucose isomerase and glucose-6-P dehydrogenase and 4 mM glucosamine-6-P (Sigma). Other enzymes were assayed spectrophotometrically at 340 nm in a buffer containing 50 mM imidazol pH7, 0.1 M KCl, 10 mM MgCl_2_, 1 mM EDTA as follows: fructose-1-6 bisphosphatase was assayed as Gancedo and Gancedo^71^, malate dehydrogenase was measured in the direction of malate formation with 1 mM freshly prepared oxaloacetate and 0.2 mM NADH, and glutamate dehydrogenase was measured with 50 mM NH_4_Cl, 0.2 mM NADH and 2.5 mM freshly prepared 2-ketoglutarate. Aconitase was assayed at 240 nm with 50 mM potassium phosphate pH 7.4 and 50 mM citrate. Fumarase activity was determined at 240 nm with 100 mM phosphate buffer pH7.6, 1 mM EDTA and 50 mM L-malate. All assays were carried out at 30 °C. Enzyme activity is expressed as milliunits/mg protein (nmol/min/mg protein). Protein was assayed with the BCA protein assay kit (Pierce).

### Statistical analysis

The data corresponding to the specific enzymatic activities were obtained from five independent experiments. Differences between values of activities in glucose and glucosamine grown cultures were examined using one-tailed, unpaired Student’s t test. A value of p < 0.05 was considered statistically significant.

### Determination of glucosamine, ammonium and ethanol

Glucosamine was assayed spectrophotometrically at 340 nm coupling the production of ADP in its phosphorylation by hexokinase to the oxidation of NADH in 0.1 M Tris-HCl pH 7.5, 2 mM ATP-Mg, 0.3 mM NADH, 2 mM PEP, and excess pyruvate kinase and lactate dehydrogenase. Ammonium was assayed spectrophotometrically at 340 nm following the oxidation of NADPH in a mixture consisting of 0.1 M triethanolamine pH8, 0.2 mM NADPH, 10 mM α- ketoglutarate and 2 units of NADPH dependent glutamate dehydrogenase (NZYTech, Lisbonne, Portugal). Ethanol was assayed in supernatants of culture samples taken along time using the Ethanol AK00061 kit from NZYTech.

### Cell growth and dry weight determination

Growth was followed by readings of optical density at 600 nm. Dry weight was determined by vacuum filtering known volumes of cultures through pre-weighed Millipore filters AAWPO4700. After drying at 100 °C for 15 hours and returning to room temperature in a desiccator the residue was weighed.

## Electronic supplementary material

Table S1
