# Supplementary material for: Construction and characterization of a Saccharomyces cerevisiae strain able to grow on glucosamine as sole carbon and nitrogen source
Source: Sci Rep. 2018 Nov 16;8:16949. doi: 10.1038/s41598-018-35045-8 (PMC6240059; doi:10.1038/s41598-018-35045-8)
Supplement: Supplementary file 1 — Table S1 [file 41598_2018_35045_MOESM1_ESM.docx]

Construction and characterization of a *Saccharomyces cerevisiae* strain able to grow on glucosamine as sole carbon and nitrogen source

Carmen-Lisset Flores and Carlos Gancedo

| Primer Name | Sequence |
| --- | --- |
| YlNAG1-F | ACCCACCAGCACAATGATCC |
| YlNAG1-R | TTCACTTGGCCTCGACCTTC |
| HXT1-F | TGGATCCAAATCATGAATTCAACTCCCGATC |
| HXT1-R | CGTCGACAGTTTATTTCCTGCTAAACAAACTC |
| HXT2-F | TGGATCCGCAACATAATGTCTGAATTCGCTAC |
| HXT2-R | CGTCGACTAATCTCTTATTCCTCGGAAACTC |
| HXT3-F | TGGATCCCAATCATGAATTCAACTCCAG |
| HXT3-R | CGTCGACGTGAAATTATTTCTTGCCGAAC |
| HXT3-Fa | CCATCACCATCACACTAGTGGATCCCAATCATGAATTCA |
| HXT3-Ra | TAACTAATTACATGACTCGAGGTCGACGTGAAATTATTTCTTGCC |
| HXT4-F | TGGATCCGCCAAAAATGTCTGAAGAAGCTGC |
| HXT4-R | CGTCGACACTGACCTACTTTTTTCCGAAC |
| HXT5-F | CCCCGGGGAAAGAATGTCGGAACTTGAA |
| HXT5-R | CGTCGACGAGATTATTTTTCTTTAGTGAACATCC |
| HXT6/7-F | TGGATCCAATGTCACAAGACGCTGCTA |
| HXT6/7-R | CGTCGACCGCAAATTATTTGGTGCTGAAC |
| HXT6/7-Fb | CACCATCACCATCACACTAGTGGATCCAATGTCACAAGACG |
| HXT6/7-Rb | CTAATTACATGACTCGAGGTCGACCGCAAATTATTTGGTGCTG |

Table S1. Primers used for PCR reactions. Since *HXT6* and *HXT7* genes differ only in 3 bp internal to the ORF the same primers were used to clone both genes.
